# A novel surgical approach with peritonectomy to extranodal multisystemic histiocytic sarcoma: A case report and literature review^☆^

**DOI:** 10.1016/j.ijscr.2019.05.027

**Published:** 2019-05-22

**Authors:** Giuseppe Barbato, Alessandro Tarantini, Francesco Serra, Francesca Cabry, Alberto Farinetti, Lorena Sorrentino, Anna Vittoria Mattioli, Roberta Gelmini

**Affiliations:** Department of Surgery, University of Modena and Reggio Emilia – Policlinico of Modena, Via del Pozzo, 71 41100, Modena, Italy

**Keywords:** Histiocytic sarcoma, Peritonectomy, Peritoneal metastases

## Abstract

**Introduction:**

Histiocytic sarcoma (HS) is a very rare malignant histiocytic derivation cancer. The extranodal multisystemic HS has an aggressive clinical course and poor Disease-Free Survival (DFS) and Overall Survival (OS). There are no shared and effective therapeutic protocols; our approach aims to improve the prognosis for advanced diseases.

**Presentation of the case:**

53-year-old female patient admitted to hospital for intestinal obstruction in April 2016 with evidence of an ileal mass in the right iliac fossa and peritoneal metastases, undergoing urgent surgery of ileal resection, ileostomy and nodular peritoneal mass biopsy. Histological examination diagnosed HS. Staging exams excluded the involvement of other gastro-intestinal districts in the absence of concomitant lymphoproliferative disorders, and PET-CT revealed multiple abdominal and mediastinal nodes hyperplasia. The patient performed chemotherapy and, seeing the partial abdominal extranodal response, was performed cytoreductive surgery (CRS). Histological examination confirmed the diagnosis of HS with multiple peritoneal, ileal, colic and omental localisation. Adjuvant second-line chemotherapy treatment (4 DHAP cycles) was performed. Disease Free Survival at ten months from cytoreductive surgery, Overall Survival at 21 months from diagnosis.

**Discussion:**

Despite a multimodal therapy with surgery and chemotherapy, extranodal multisystemic HS has a poor prognosis. Up to now the role of surgery is limited to biopsies or complications treatment. Our results of DFS and OS show that cytoreductive surgery may be a valid therapeutic choice.

**Conclusion:**

The surgical approach with major cytoreductive purposes could improve the prognosis in cases with prevalent abdominal extranodal localisation.

## Highlights

• Cytoreductive surgery (CRS) for very rare malignant disease.
• A novel surgical approach to treat the histiocytic sarcoma in human.
• The role and the importance of the multidisciplinary approach in the decision making process for a rare disease.

## 1 Introduction

Histiocytic sarcoma (HS) is a very rare malignant neoplastic disease with only a few hundred cases reported in the literature. According to data from the SEER database of U.S. National Cancer Institute, HS is more common in adults with a median age of 63 years despite having an extremely wide range (from 18 to 96 years) with a slight incidence in favour of males (1.5:1) [1,2]. The pathogenesis is unclear, no predisposing hereditary or environmental factors are known. Unlike the other sarcomas, the origin is from microcytic-macrophage system cells expressing immunophenotypical and morphological characters of histiocytic derivation [[3], [4], [5]].

The clinicopathological manifestation could be as primitive extranodal neoplastic disease [6] or associated with malignant haematological disorders such as follicular lymphoma or acute lymphoblastic leukaemia [5,7]. The diagnosis is based on histological examination and immunohistochemical characterisation [3,8]. The clinical presentation is frequently asymptomatic, with incidental diagnosis during radiological investigations; it may occur with asthenia or with symptomatology correlated to involved surrounding organs, the most frequent localisations are small intestine, skin and soft tissues. The most common symptoms onset is the appearance of a palpable mass with associated compressive symptoms or systemic complaints such as weight loss or fever [3]. The HS has an aggressive clinical course mainly in multisystemic disease [6]. Because of HS low incidence and prevalence, in literature, there are no prospective studies. The available data came from single case reports or small case series that do not provide to elaborate on a widely shared management. Cytoreductive surgery has shown promising results in the treatment of advanced multifocal malignancy and peritoneal metastasis [[9], [10], [11], [12]].

Up to now, the role of surgery is almost confined to biopsies or complications treatment. Our results of DFS and OS show that cytoreductive surgery may be a valid therapeutic choice for improving the prognosis of advanced extranodal abdominal HS.

## 2 Presentation of the case

53-year-old female patient with a history of latent tuberculosis infection in prophylactic treatment with isoniazid, previous surgery of tonsillectomy, appendectomy and cholecystectomy. Family history negative for neoplastic diseases. Hospitalised at another institute in April 2016 with clinical manifestation of intestinal obstruction with evidence for CT scan of a solid occluding mass of a distal ileus of 5 cm maximum diameter localised in the right iliac fossa. Multiple nodular neoformations at the peritoneal level, the largest of 2 cm maximum diameter with associated free fluid in the abdomen and multiple lymphadenopathies of the ileal mesentery and some enlarged lymph nodes of the right anterior heart-phrenic angle. The latest was the unique finding of extra-abdominal disease spread. So the patient underwent surgery for ileal resection and ileostomy, with a peritoneal nodule biopsy.

**Fig. 1:**
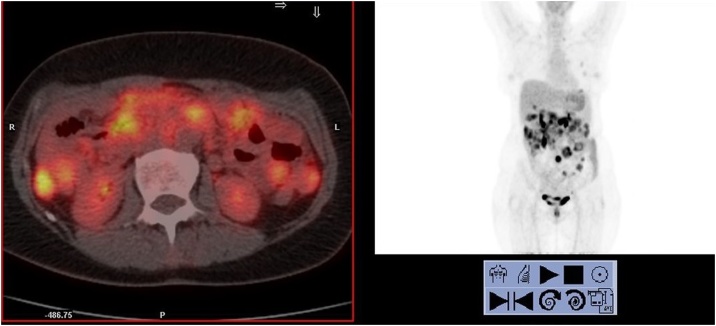
CT-PET pre adjuvant chemotherapy.

The definitive histological examination of the ileal mass (free surgical resection margins) characterization showed positive reactions for histiocytic markers (CD163 and CD68); negative reactions for lymphoid markers (CD45 / LCA, CD20 / L26, CD79a, CD3, CD2, CD5, CD7, CD8, CD56, CD30 / BERH2 and ALK1) and myeloperoxidase. The reaction for cNPM is negative. Negative reactions for cytokeratins (MoAB MNF116, CK8,18, AE1 + AE3), desmin, caldesmon, ML actin, CD34, CD117, DOG1, S100 protein, melanA, CD21, CD1a. Cytoproliferative activity (MIB1-LI) in 15–20% of cells. Biopsy histological examination on the major of the peritoneal nodularity confirms the diagnosis of HS peritoneal localisation. The patient evaluation was performed with Esophagogastroduodenoscopy and Colonoscopy that excluded the involvement of other gastrointestinal tracts, bone marrow biopsy to confirm the absence of concomitant lymphoproliferative diseases. PET-CT showed multiple uptakes at the ileal mesentery, right and left colon, lymph node uptake also at the thoracic level at the phrenic heart angle, a finding already known in previous exams. The patient then performed six cycles of CHOEP chemotherapy. After adjuvant therapy, the PET-CT described a partial response to treatment with regression of uptake areas at thoracic level and persistence of abdominal uptake of already knows localisations (Fig. 1). After a multidisciplinary evaluation, was indicated the CRS: at the exploration of the abdominal cavity was established the disease recurrence from 15 cm to ileostomy, in the pelvis and along the left colonic mesentery and parietal peritoneum until homolateral hypochondrium, near the spleen. So the decision it was to perform an ileal en-bloc resection of the previous ileostomy, total colectomy, hysterectomy and bilateral oophorectomy, bilateral obturator lymphadenectomy, omentectomy, splenectomy, peritonectomy of all four abdominal quadrants removing multiple peritoneal nodules of the ileal mesentery. The spleen was removed to guarantee the radicality of surgery considering the presence of nodular lesions on the diaphragmatic peritoneal surface where the spleen leans. Peritoneal Cancer index was 13; at the end of the procedure, Cytoreduction score was CC-0.

Post-operative course was characterised by close monitoring in ICU up to the 3rd postoperative day and then transferred to our ordinary ward, the onset of infectious pneumonia resolved with antibiotic therapy. Discharge of the patient at home on the 20th postoperative day. Histological examination confirmed the diagnosis of HS with ileal relapse, multiple peritoneal, small bowel mesentery, colic and omental localisation, negative for HS localisation the other tissues removed. The postoperative CT scan showed a complete resection of abdominal HS localisation such as CT-scan at three and six mounths-follow-up. During the same period the patient underwent a second-line chemotherapy treatment with four cycles according to DHAP scheme (Dexamethasone, Cisplatin, Aracytin). The subsequent CT-scan performed at ten months from cytoreductive surgery (17 months from diagnosis) highlighted a disease relapse with multiple abdominal and thoracic localisations. Exitus at 21 months after diagnosis due to progressive decay of the general performance status.

## 3 Discussion

The main feature of this case is the relatively long disease-free survival of the patient obtained with an innovative surgical approach. HS is an extremely aggressive neoplastic disease for which, as a second-line treatment after partial response to adjuvant chemotherapy, it was chosen, after multidisciplinary discussion, to surgical cytoreductive treatment. There is no standardise therapy for HS, the rarity and consequently, the smallness data present in literature does not allow the formulation of a widely shared diagnostic and therapeutic management. After pathologic evaluation with the exclusion of other pathologies of histiocytic aetiology by immunohistochemistry, for the patient was suggested cytoreductive surgery. The correct procedure of CRS consists of being as radical as possible, so it was necessary to make the total colectomy, the oophorectomy and the splenectomy. The spleen was removed to guarantee the radicality of surgery. In literature, data on OS and DFS for HS with multisystemic abdominal localisation are few and fragmented. Pileri conducted one of the major reviews in 2002 [5] which analyses 61 cases. Selecting among the cases in the review we have found four patients (6.5% of analysed HS) with disseminated extranodal HS (cfr: Cases number 7-8-10-16 with stage > III) all treated with chemotherapy alone; none of these reaches the first follow-up step for DOD (death due to the disease). Another important study [13] analyses histiocytes or dendritic cells sarcoma treated at Memorial Sloan Kettering Cancer Center between 1995 and 2014. From the study arisen only four cases with disseminated extranodal HS and there are not stratified enough data to be able to extrapolate useful cases for comparison. Detailed literature research did not identify case reports or other studies that show correspondence with the illustrated case.

HS is an extremely aggressive neoplastic disease for which in our centre, as a second-line treatment after partial response to adjuvant chemotherapy, it was chosen, after multidisciplinary discussion, to surgical cytoreductive treatment a noticeable result when compared to the poor prognosis of multisystem extranodal cases.

The limited knowledge of the HS natural history makes every report precious. Development of clinical trials that allow the elaboration of a widely shared therapeutic, diagnostic procedure is needed. In such rare diseases, randomised or prospective studies are hard to achieve, and case reports will continue to be the foundation management of these patients.

## 4 Conclusion

Proper integration between oncologists and haematologists, radiologists and surgeons resulted in the correct management of a patient suffering from a rare disease. The authors suggest, in well-selected patients and after the tumour board discussion, to consider CRS as another therapeutical option in this aggressive disease, ever after adequate chemotherapy that, to date, must be considered the first line of therapy ever.

Further studies are needed to validate the hypothesis and broaden the knowledge of this rare disease.

## Conflicts of interest

No conflicts of interest.

## Sources of funding

Non funding were used.

## Ethical approval

The ethical approval for this case report is been exempt

The submitted case report was not a study, therefore no ethical approval or informed written consent was needed

## Consent

Written informed consent was obtained from the patient for publication of this case report and accompanying images. A copy of the written consent is available for review by the Editor-in-Chief of this journal on request

## Author contribution

Barbato Giuseppe, MD - Data collection and author of case report and discussion.

Tarantini Alessandro, MD - Data collection and author of case report and discussion.

Serra Francesco, MD -Review of surgical technique literature and co-author of discussion.

Cabry Francesca, MD - Review of surgical technique literature and author of introduction.

Farinetti Alberto, MD - Review of literature and co-author.

Sorrentino Lorena, MD - Review of surgical technique literature and author of introduction.

## Registration of research studies

The submitted case report is not a research study.

## Guarantor

Gelmini Roberta.

## Disclosure statement

The authors have nothing to disclose.

## Provenance and peer review

Not commissioned, externally peer-reviewed.
